# 2507: Towards a scalable informatics platform for enhancing accrual into clinical research studies

**DOI:** 10.1017/cts.2017.84

**Published:** 2018-05-10

**Authors:** Ram Gouripeddi, Elizabeth Lane, Randy Madsen, Ryan Butcher, Bernie LaSalle, Katherine Sward, Julie Fritz, Julio C. Facelli, Mollie Cummins, Jianyin Shao, Rob Singleton

**Affiliations:** The University of Utah School of Medicine, Salt Lake City, NY, USA

## Abstract

OBJECTIVES/SPECIFIC AIMS: Issues with recruiting the targeted number of participants in a timely manner often results in underpowered studies, with more than 60% of clinical studies failing to complete or requiring extensions due to enrollment issues. The objective of this study is to develop and implement a scalable, organization wide platform to enhance accrual into clinical research studies. METHODS/STUDY POPULATION: We are developing and evaluating an informatics platform called Utah Utility for Research Recruitment (U2R2). U2R2 consists of 2 components: (i) Semantic Matcher: an automated trial criterion to patient matching component that also reports uncertainty associated with the match, and (ii) Match Delivery: mechanisms to deliver the list of matched patients for different research and clinical settings. As a first step, we limited the Semantic Matcher to utilize only structured data elements from the patient record and trial criteria. We are now including distributional semantic methods to match complete patient records and trial criteria as documents. We evaluated the first phase of U2R2 based on a randomized trial with a target enrollment of 220 participants that compares 2 treatment strategies for managing back pain (physical therapy and usual care) for individuals consulting a nonsurgical provider and symptomatic <90 days. RESULTS/ANTICIPATED RESULTS: U2R2 identified 9370 patients from the University of Utah Hospitals and Clinics as potential matches. Of these 9370, 1145 responded to the Back Pain study research team’s email or phone communications, and were further screened by phone. In total, 250 participants completed a screening visit, resulting in the current study enrollment of 130 participants. Forty-three of 1145 patients refused to participate, and 50 participants no-showed their screening visit. DISCUSSION/SIGNIFICANCE OF IMPACT: A recruitment platform can enhance potential participant identification, but requires attention to multiple issues involved with clinical research studies. Clinical eligibility criteria are usually unstructured and require human mediation and abstraction into discrete data elements for matching against patient records. In addition, key eligibility data are often embedded within text in the patient record. Distributional semantic approaches, by leveraging this content, can identify potential participants for screening with more specificity. The delivery of the list of matched patient results should consider characteristics of the research study, population, and targeted enrollment (eg, back pain being a common disorder and the possibility of the patient visiting different types of clinics), as well as organizational and socio-technical issues surrounding clinical practice and research. Embedding the delivery of match results into the clinical workflow by utilizing user-centered design approaches and involving the clinician, the clinic, and the patient in the recruitment process, could yield higher accrual indices.

